# The QuantuMDx Q-POC SARS-CoV-2 RT-PCR assay for rapid detection of COVID-19 at point-of-care: preliminary evaluation of a novel technology

**DOI:** 10.1038/s41598-023-35479-9

**Published:** 2023-06-17

**Authors:** Jessica Caffry, Matthew Selby, Katie Barr, George Morgan, David McGurk, Philip Scully, Catherine Park, Anna-Maria Caridis, Emily Southworth, Jack Morrison, David J. Clark, Benedict M. O. Davies, Nicholas M. Eckersley, Elisabetta Groppelli, Daniela E. Kirwan, Irene Monahan, Yolanda Augustin, Colin Toombs, Tim Planche, Henry M. Staines, Sanjeev Krishna

**Affiliations:** 1 QuantuMDx, Lugano Building, 57 Melbourne St, Newcastle Upon Tyne, UK; 2 Clinical Academic Group in Institute for Infection and Immunity, St George’s University of London, London, UK; 3 St George’s University Hospitals NHS Foundation Trust, London, UK; 4 Institut für Tropenmedizin, Universitätsklinikum Tübingen, Tübingen, Germany; 5 Centre de Recherches Médicales de Lambaréné, Lambaréné, Gabon

**Keywords:** Viral infection, PCR-based techniques

## Abstract

Accurate and rapid point-of-care (PoC) diagnostics are critical to the control of the COVID-19 pandemic. The current standard for accurate diagnosis of SARS-CoV-2 is laboratory-based reverse transcription polymerase chain reaction (RT-PCR) assays. Here, a preliminary prospective performance evaluation of the QuantuMDx Q-POC SARS-CoV-2 RT-PCR assay is reported. Between November 2020 and March 2021, 49 longitudinal combined nose/throat (NT) swabs from 29 individuals hospitalised with RT-PCR confirmed COVID-19 were obtained at St George’s Hospital, London. In addition, 101 mid-nasal (MN) swabs were obtained from healthy volunteers in June 2021. These samples were used to evaluate the Q-POC SARS-CoV-2 RT-PCR assay. The primary analysis was to compare the sensitivity and specificity of the Q-POC test against a reference laboratory-based RT-PCR assay. The overall sensitivity of the Q-POC test compared with the reference test was 96.88% (83.78– 99.92% CI) for a cycle threshold (Ct) cut-off value for the reference test of 35 and 80.00% (64.35–90.95% CI) without altering the reference test’s Ct cut-off value of 40. The Q-POC test is a sensitive, specific and rapid PoC test for SARS-CoV-2 at a reference Ct cut-off value of 35. The Q-POC test provides an accurate option for RT-PCR at PoC without the need for sample pre-processing and laboratory handling, enabling rapid diagnosis and clinical triage in acute care and other settings.

## Introduction

Severe acute respiratory syndrome coronavirus 2 (SARS-CoV-2) is an enveloped, positive sense, single-stranded RNA virus of the genus betacoronavirus that caused the corona virus disease 2019 (COVID-19) pandemic with devastating public health impact. By the end of 2022, there have been reported more than 650 million individuals infected and more than 6.5 million deaths globally^1^. The first diagnostic assays for this disease were developed shortly after the initial publication of the viral genome in January 2020^2^. These were nucleic acid amplification tests (NAATs) based on reverse transcriptase polymerase chain reaction (RT-PCR), where target viral RNA sequences are converted to DNA and exponentially amplified to allow detection with fluorescent probes. The lower the concentration of target RNA sequence in a clinical sample, the higher the cycle threshold (Ct) value at which fluorescence is detectable.

Most convenient assays use upper respiratory (*e.g.* nasopharyngeal or oropharyngeal or combined nose/throat (NT)) swab samples to detect SARS-CoV-2 RNA and are highly sensitive^3^. Viral load is highest in the upper respiratory tract in the first week of illness^4^. However, standard laboratory-based RT-PCR assays are relatively complex and time consuming, requiring highly trained personnel and expensive equipment based in a centralised laboratory. They take several hours to perform in batches, and, when time is added for sample transport, results often take 24 h or more to be reported. This can result in delayed diagnosis, which can complicate clinical triage.

Point-of-care (PoC) diagnostics were identified as one of eight key research priorities to tackle the pandemic^5^. Rapid, PoC or near-patient testing for active infections confers several benefits over laboratory-based testing^6–8^ including faster triage, which in turn aids infection control and clinical management. Rapid PoC tests can also enable effective community testing and alleviate pressure on overburdened centralised labs. While lateral flow-based rapid antigen tests (RDTs) are now in widespread use, they are less sensitive than NAATs^9^. Thus, development of rapid PoC NAAT-based tests are a priority, especially for use when immediate actions are needed for positive cases such as in accident and emergency departments, pre-surgical or chemotherapy day unit admissions, care homes, airports, prisons and low resource healthcare settings. Several versions of PoC NAATs have been developed^10–13^, but all have one or more limitations including long sample-to-result times, complexity (steps required) and cost.

To address the urgent need for a rapid and sensitive COVID-19 diagnostic, QuantuMDx have repurposed their RT-PCR PoC diagnostics platform, the Q-POC, to run a SARS-CoV-2 assay. Here, we present a detailed description of this technology and a preliminary prospective performance evaluation of Q-POC compared with validated laboratory-based RT-PCR assays.

## Methods

### Ethics statement

The study was conducted in accordance with relevant UK guidelines and regulations. Ethical approval for clinical samples collected at St George’s Hospital was provided by the Institutional Review Board (Integrated Research Application System project ID: 282104; South Central—Oxford C Research Ethics Committee reference: 20/SC/0171; registered at clinicaltrials.gov NCT04351646) as part of the “Development and Assessment of Rapid Testing for SARS-CoV-2 outbreak” (DARTS) study, sponsored by St George’s Hospital NHS Foundation Trust. The samples collected at QuantuMDx were from healthy individuals undergoing testing in a pre-existing staff COVID-19 testing programme. The samples were collected for the purposes of determining test performance (test performance evaluation) and were anonymised, therefore ethical approval was not deemed necessary. All participants for this study were recruited following informed consent.

### Swab samples

Patients with COVID-19 hospitalised at St George’s Hospital were recruited into the prospective study between 19th November 2020 and 3rd March 2021 (inclusive). Prior to recruitment, they were confirmed as positive for SARS-CoV-2, following NT swabs (in Sigma Virocult, Corsham, UK) taken as standard of care at hospital admission. These were processed on site at South West London Pathology, using Roche RNA extraction kits (Magnapure, West Sussex, UK) followed by RT-PCR with altona Diagnostics RealStar SARS-CoV-2 RT-PCR (S and E target genes, Hamburg, Germany) or Roche cobas SARS-CoV-2 Test (E and ORF target genes) kits. All patients consented to NT swabs being taken. Following recruitment to DARTS, longitudinal NT swab samples were collected, using a Copan swab (Cat number: 503CS01), at days 0, 3, 5, 7, 10 and 14 (± 2 days) or until they withdrew from the study. Anonymised samples were transferred to long-term storage within 2 h of collection on the wards, without viral transport medium, at -80 C until use. In addition, anonymised mid-nasal (MN) swab samples were collected between 15 and 18th June 2021 (inclusive) from healthy staff volunteers at QuantuMDx following informed consent and tested immediately.

### Q-POC testing

**Figure 1:**
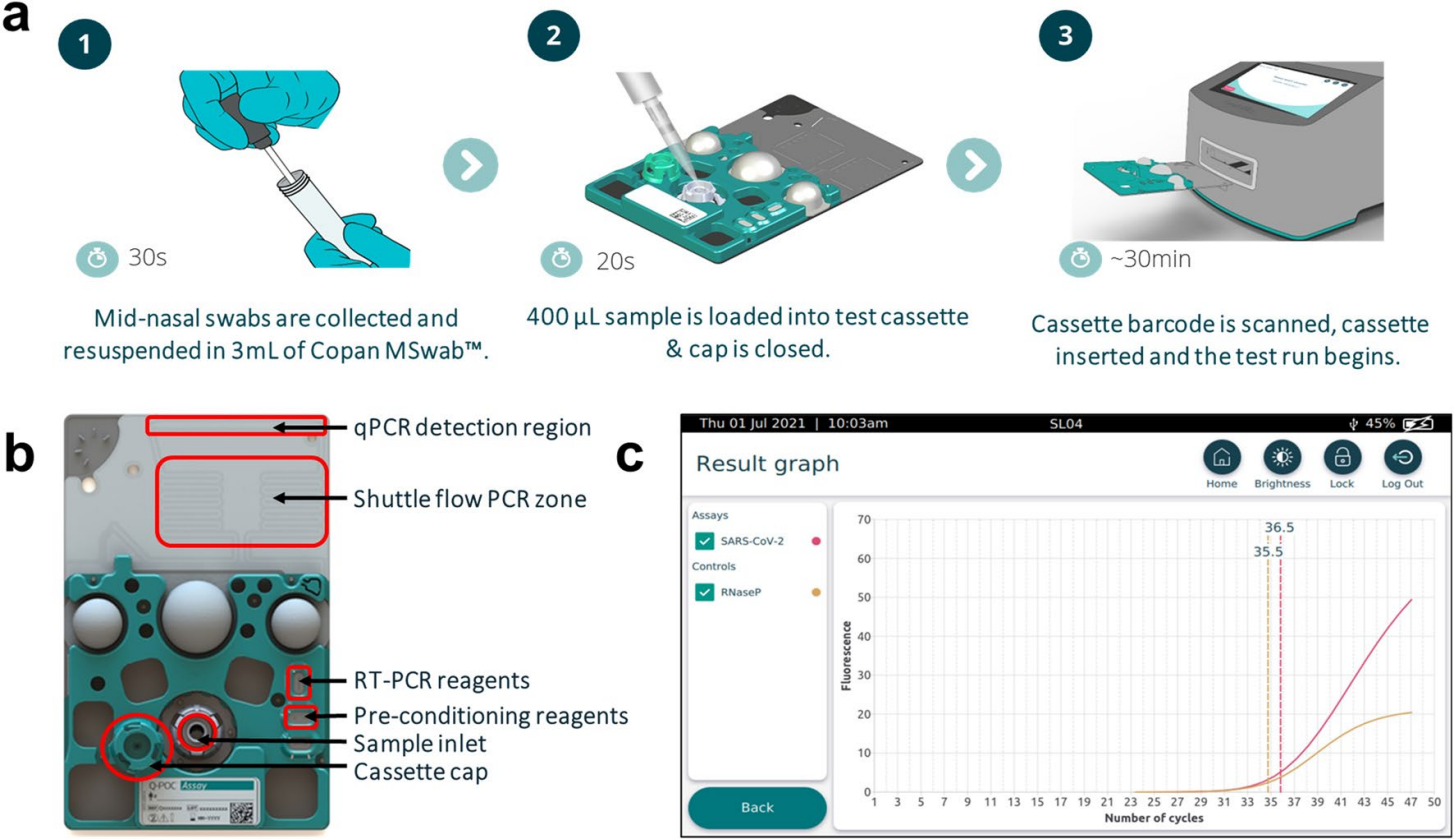
QuantuMDx Q-POC and Q-CAS, point-of-care diagnostic for detection of SARS-CoV-2 from mid-nasal (MN) swab samples. (**A**) Graphic showing the Q-POC/Q-CAS workflow. A MN swab is collected and resuspended in Copan MSwab (3 ml). The tube is inverted 5 times before 400 µl is dispensed into the inlet of the cassette using a pipette. The barcode is scanned, Q-POC identifies the cassette, the cassette is inserted and Q-POC begins the SARS-CoV-2 assay. A result is displayed on screen in ~ 30 min. (**B**) Key areas of the Q-CAS cassette. Q-POC interacts with the self-contained cassette to move the sample from the inlet through the lyophilised reagents and into the shuttle flow PCR region. RT-PCR amplification is detected during shuttling through the detection region. (**C**) Results graph for a positive SARS-CoV-2 sample. The assay results in PCR curves for SARS-CoV-2 (in red) and sample control, RNase P (in yellow). This screen is optionally displayed after the main results screen whereby a definitive result is displayed (positive/negative/invalid). Results can be reviewed, printed and uploaded.

The Q-POC device is a PoC diagnostics platform, which runs a rapid molecular test in a single use microfluidic cassette (Q-CAS) by incorporating sample processing, DNA amplification (with RT step, if required), and downstream qualitative detection of pathogens in small volumes of sample (Fig. 1). The total hands-on time is approximately one minute. It involves two main steps, (1) loading a 400 µl sample into a test cassette and (2) inserting the test cassette into the instrument. Results can be interpreted after ~ 30 min.

For detection of SARS-CoV-2 by RT-PCR at PoC a flexible mini tip Copan FLOQSwab (designed for nasopharyngeal sampling) was used to take a NT or a MN swab. The tip (fresh or defrosted) was then broken off into a sample tube containing 3 ml of MSwab sample collection, transport and preservation medium (Copan Diagnostics, Italy). Following sample tube inversion (× 5), a 400 µl aliquot is pipetted into a test cassette, Q-CAS, and sealed in with a cap.

The loaded cassette is then scanned and loaded into the Q-POC device. Within the Q-CAS, reconstitution of lyophilised RT-PCR reagents, held within sealed ‘Reagent Fuses’ for increased shelf-life, occurs using 50 µl of the swab eluate containing released RNA. Following an RT step, rapid PCR is achieved by shuttling fluid between heating zones (shuttle flow PCR, sfPCR—Fig. 1B). This allows target amplification and detection in 32 min and equates to 48 cycles (with a threshold for positive samples of ≤ 45). SARS-CoV-2 gene targets (Orf1ab, N and S) are all detected in a single fluorescence channel (FAM), while an RNase P target (used as a control) is detected in a second channel (HEX). Fluorescence readings are taken after each cycle (qPCR reader) and are analysed to provide a qualitative result. The use of individual heating zones combined with shuttling of the fluid, unlike the temperature ramping used by conventional PCR, is far more energy efficient allowing for multiple assay runs even when the Q-POC is battery operated. The Q-POC is front-loading and intuitive, with a simple touch screen interface designed for PoC testing in resource limited settings. It contains all the necessary mechanics, electronics and optics to drive the cassette-based assays, in addition to reading, interpreting and presenting test results. With regards to analysis of data, a calling algorithm gives a positive, negative or invalid result with no need for user interpretation of curves. It is portable and operable either on mains power or by an exchangeable battery, which can last up to 4 h. Data are recorded and initially stored on the Q-POC within a secured database, with a storage capacity of > 1000 patient results. In addition, the Q-POC will connect to a specified receipt-style printer. The Q-POC operating system runs on an ARM-based processing board integrating connectivity options: GPS, Bluetooth, Wi-Fi and GPRS (2G/3G) connectivity. Operation data are also retained within the Q-POC to allow for tracking and trending of instrument performance.

### Laboratory-based reference testing

An aliquot (140 µl) of each swab sample in 3 ml of MSwab was used with a QIAamp Viral RNA Mini Kit (Qiagen, Germany) for RNA extraction, as per the manufacturer’s instructions. The CE IVD-marked SARS-CoV-2 RT-PCR Detection Assay (QuantuMDx, UK) was used as the reference test and gold standard for assessing the Q-POC SARS-CoV-2 RT-PCR assay. It is based on TaqMan chemistry and utilises FAM for the detection of the three SARS-CoV-2 loci, Orf1ab, N gene and the S gene and HEX for the detection of the RNase P gene (a specimen and process control). The assay takes approximately 75 min to run, undertaking 45 cycles of PCR. The cycle threshold (Ct) for detection of all SARS-CoV-2 targets is 40, with ≤ 40 indicating a valid positive result. The Ct for detection of the RNase P target is 35, with ≤ 35 with no SARS-CoV-2 signal (or Ct > 40) indicating a valid negative result. Runs are invalid if there is no SARS-CoV-2 signal (or Ct > 40) and no RNase P signal (or Ct > 35).

### Statistical analysis

Operators were not blinded to the status of patients/volunteers from which the samples were taken prior to Q-POC testing but did not have access to the results of the subsequent reference testing; however, the Q-POC requires no subjective interpretation of a result.

Data were analysed with GraphPad Prism (version 6.07 for Windows). Diagnostic sensitivity, specificity, positive predictive value and negative predictive value were determined, using the laboratory-based RT-PCR as a reference standard (Supplementary Data S1). Samples that were invalid on the PoC platform were not included in the primary sensitivity analysis. The manuscript was prepared in accordance with the EQUATOR Network’s STARD guidelines (Supplementary Data S2).

## Results

**Figure 2:**
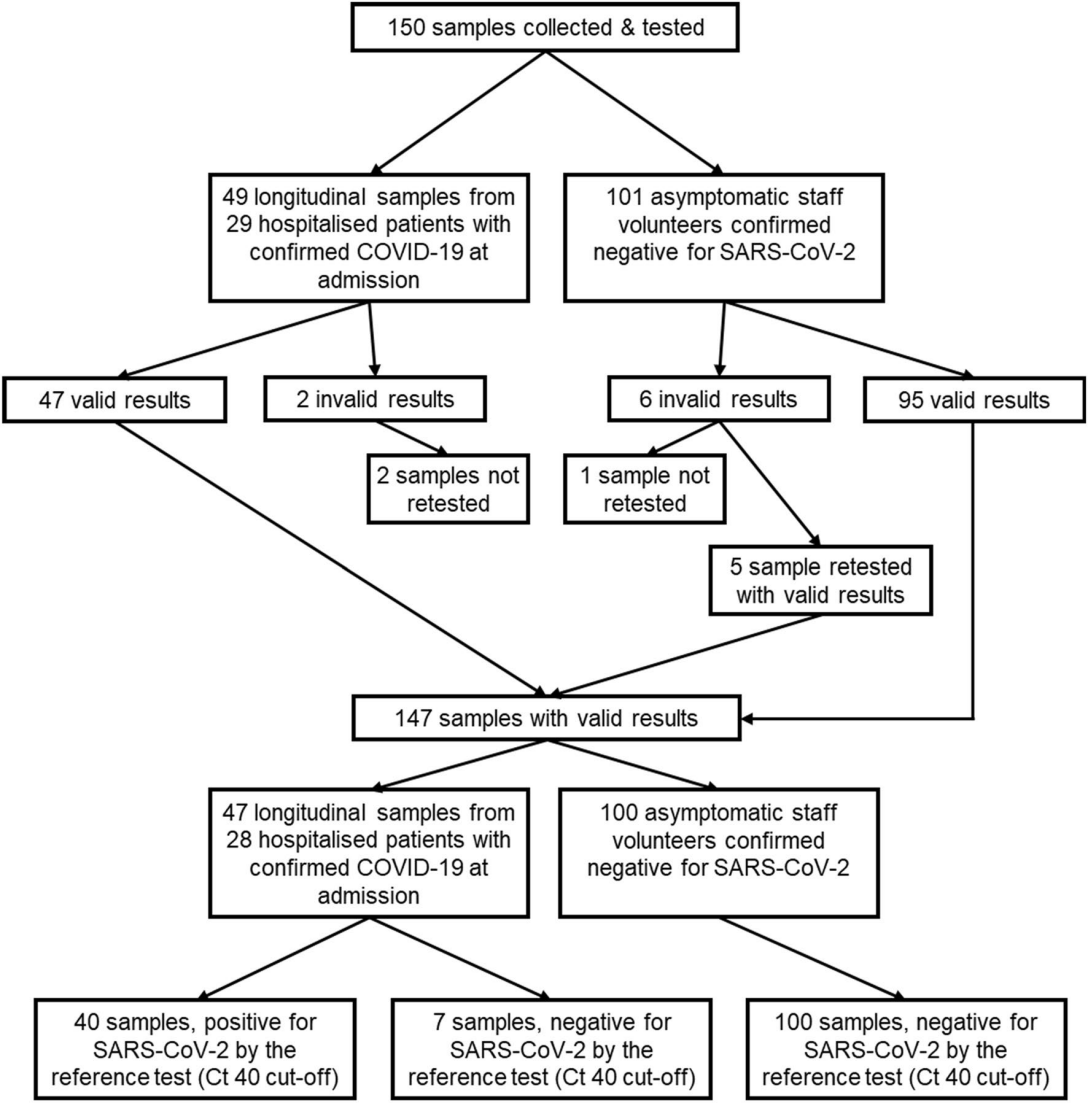
Study design.

Swab samples (n = 150) were collected and tested, using the Q-POC (Fig. 2). Two cohorts were recruited. In the first, hospitalised patients at St George’s Hospital with an RT-PCR confirmed COVID-19 diagnosis at admission were clinically assessed between the 19^th^ November 2020 and 3rd March 2021 as part of DARTS. The recruits provided longitudinal samples predicted to have a wide range of viral loads thereby providing a challenging sample set for initial evaluation of the Q-POC. Patients (n = 29) who provided a NT sample, using a mini tip Copan swab, were eligible for inclusion in the Q-POC evaluation. The median age of COVID-19 positive study participants included in the study was 64 (IQR 52–74), 20 (69%) were male, 16 (55%) were symptomatic and the majority (24; 83%) had mild disease at enrolment (Table 1).

**Table 1:** Patient demographics.

Demographic	N (%)
Total number of patients included in study	29
Male sex	20 (69%)
Median age (years)	64
Symptomatic at time of study enrolment	16 (55%)
WHO ordinal symptom severity scale (OSSS) baseline enrolment^a^	WHO OSSS 4 = 24 (83%) WHO OSSS 5 = 4 (14%) WHO OSSS 6 = 1 (3%)

^a^WHO OSSS 4 = Hospitalised, mild disease, no oxygen therapy; WHO OSSS 5 = Hospitalised, oxygen mask or nasal prongs; WHO OSSS 6 = Hospitalised, non-invasive ventilation/high flow oxygen.

Two samples from this cohort produced invalid results on the Q-POC test and were not retested. One of these samples came from a patient who provided only one sample so only their invalid result was included in the analysis (Fig. 2). Therefore, complete clinical data paired with laboratory reference test data were available for 28 patients and 47 samples, with 40 samples testing positive and 7 testing negative using the reference test (along with the specified Ct cut-off of 40). Participants formed a convenience series. No adverse events from performing the Q-POC or the reference test were recorded.

In the second cohort, 101 MN Copan swab samples were collected from healthy volunteer staff to provide a SARS-CoV-2 negative dataset. Of these, 6 samples returned an invalid Q-POC test result. One was not retested and 5 were retested, producing valid results. Therefore, test data for further analysis were available for 100 samples from this cohort, all of which tested negative with the reference test (Fig. 2).

Overall, a total of 155 sample runs between the 2 cohorts were undertaken on the Q-POC. Of these, there were 8 invalid runs, 5 of which were retested to produce valid results, and 3 of which were not retested as the samples were destroyed (Fig. 2). This gives an invalid rate over the study period of 5.2%. Of the 147 samples with paired Q-POC and reference test data, 40 were positive and 107 negative for SARS-CoV-2 with the reference test.

**Figure 3:**
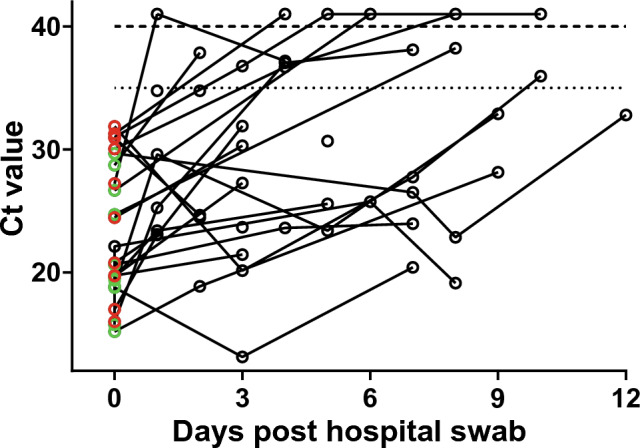
Longitudinal SARS-CoV-2 Ct values for COVID-19 patient samples. The hospital Ct results that were available are presented as the E gene Ct values for either the Roche (red symbols) or altona (green symbols) assays and used as day 0. Other data points are Ct values from the laboratory-based SAR-CoV-2 RT-PCR reference assay (black symbols). Any data points above the dashed line are negative and have nominally been given a Ct value of 41 to allow presentation. A Ct cut-off of 35 is represented by the dotted line. Note that both of the RT-PCR assays used by St George’s Hospital here have two independently measured targets, one of which is the E gene. The Ct values for the E gene target only were chosen to be presented since the average difference in Ct values between the E gene and the other target within each assay used were − 0.24 ± 0.29 (mean ± SEM; n = 10; range − 2.2 to 0.45) and 0.48 ± 0.11 (mean ± SEM; n = 12; range 0–1) for the Roche and altona assays, respectively.

Figure 3 presents the Ct values from the reference test used with longitudinal NT swab samples from COVID-19 positive participants. As predicted, this sample set provided a wide range of viral loads (using Ct values as a basic proxy), including multiple samples between Ct values of 30 and 40. Included in the graph are the available Ct data from the hospital test undertaken prior to enrolment (for 22 of the 28 participants analysed). The day the swab was taken for this test was used as day 0. Two different validated assays (see “Methods”) were used at St George’s Hospital over the study period. Irrespective of the test used, there is a general increase in Ct values with time after the first test. No participants with a hospital test Ct value below 26 produced a negative result within the short (12 day) study period, while several participants with a starting hospital Ct value above 26 were negative at subsequent time points.

**Figure 4:**
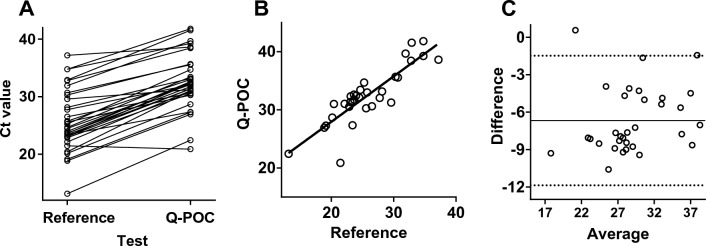
Comparison between Ct values generated by the laboratory-based SAR-CoV-2 RT-PCR reference assay and the Q-POC assay. (**A**) Ct values for SARS-CoV-2 positive samples called by both the reference and Q-POC tests (n = 32) with Ct values below the 40 and 45 assay cut-off values, respectively. (**B**) Linear regression plot to show the correlation be-tween the reference and Q-POC assay Ct values. The line of best fit equation is y = 0.78x + 12.30 (r^2^ = 0.76). (**C**) Bland–Altman plot to evaluate the agreement between the reference and Q-POC assay Ct values. The bias between the reference and the Q-POC test is − 6.7 Ct units (solid line) and the 95% limits of agreement are denoted by the dotted lines.

Prior to the reference RT-PCR testing undertaken to derive the data presented in Fig. 3, samples were run on the Q-POC SARS-CoV-2 RT-PCR assay. While the assay is designed to generate a SARS-CoV-2 positive or negative result, Ct values can also be derived. Figure 4 compares Ct values for the reference versus the Q-POC test. Data are from samples that were called positive by both tests (n = 32) and thus have paired Ct data. The two datasets passed normality tests (P = 0.86 and P = 0.69 for the reference and Q-POC tests, respectively; D’Agostino & Pearson omnibus normality test). Interestingly, there is very good concordance between the two paired datasets (as judged by eye, Fig. 4A). Linear regression analysis of Q-POC and reference datasets (Fig. 4B) show a strong correlation (r^2^ = 0.76). A Bland–Altman plot demonstrated very good agreement between the 2 tests, with a bias of − 6.7 Ct units meaning the Q-POC test averages a 6.7 higher Ct value with mean (± SEM) Ct values of 25.8 ± 0.9 versus 32.5 ± 0.9 for the reference and Q-POC tests respectively (Fig. 4C). This measured bias is consistent with the Ct cut-off value of 40 (above which a result is called negative) for the reference assay compared with the Ct cut-off value of 45 for the Q-POC assay.

With the inclusion of data from the 47 samples from COVID-19 patients and from single samples from 100 healthy volunteers, the performance characteristics of the Q-POC test were determined (Table 2). The overall sensitivity (95% CI) of the Q-POC test was 80.0% (64.4–91.0%) against the SARS-CoV-2 RT-PCR laboratory reference test with a specificity and 99.1% (94.9–100%), with PPV and NPV of 97.0% and 93.0%, respectively. Interestingly, the sensitivity of the Q-POC test was 96.9% (31/32) at a reference test Ct ≤ 35 and only 12.5% (1/8) at a reference test Ct > 35. Thus, if the reference test Ct cut-off was adjusted to 35, sensitivity increased significantly to 96.9% (83.8–99.9%), while specificity dropped only slightly to 98.3% (82.2–99.9%).

**Table 2:** Q-POC test performance.

Cut-off (Ct)	Total	TP	TN	FP	FN	Sensitivity (95% CI)	Specificity (95% CI)	NPV (95% CI)	PPV (95% CI)
< 40	147	32	106	1	8	80.0% (64.4–91.0)	99.1% (94.9–100)	93.0% (87.7–96.1)	97.0% (81.9–99.6)
< 35	147	31	113	2	1	96.9% (83.8–99.9)	98.3% (93.9–99.8)	99.1% (94.3–99.9)	93.9% (79.7–98.4)

*TP* true positives, *TN* true negatives, *FP* false positives, *FN* false negatives, *PPV* positive predictive value, *NPV* negative predictive value, *CI* confidence interval.

Of note, while not evaluated in this study, the instructions for use (IFU; Supplementary Data S3) for the Q-POC test states that the limit of detection is 1000 genome equivalent copies per ml with a standard deviation of 0.8 and average Ct of 41.89, which is in line with the results presented above. Furthermore, in vitro analysis for exclusivity or cross-reaction was undertaken with no cross-reactivity observed under the tested conditions. All micro-organisms were used at a concentration of 1 × 10^5^ colony forming units (cfu) per reaction. Finally, as recommended by the Medicines & Healthcare products Regulatory Agency (MHRA), UK, QuantuMDx continuously monitor and assess the assay design in silico for newly emerging variants of SARS-CoV-2 with regular reports which are published by the MHRA.

## Discussion

In a pandemic that causes a Public Health Emergency of International Concern, one of the earliest interventions is accurate diagnosis of an infection. At the start of a pandemic, this will depend largely on clinical evaluations (including imaging, biochemical and haematological assessments) that guide immediate containment measures. NAATs can be developed with urgency to diagnose a highly transmissible infectious disease accurately and assist in managing patients and breaking transmission chains (as happened with COVID-19^2^). Accurate diagnostics are also critical to support the development of new therapeutic interventions, and to assess public health measures to contain infections, including the development and implementation of vaccination programmes.

There are several challenges for developers of diagnostics in the early stages of a pandemic. Regulatory authorities will be as unfamiliar with a disease such as COVID-19 as are other experts and this may delay appropriate evaluation of tests under development. Urgent need coupled with limited supplies and a motive to profit can encourage the proliferation of diagnostic tests that are easy to produce in bulk (such as lateral flow assays) but that fail to meet minimum requirements for accuracy (even if these requirements have been agreed, which itself can take too long procedurally). One way to circumvent some of these limitations is to repurpose high quality NAAT-based testing platforms to deal with new infections such as those caused by SARS-CoV-2.

The COVID-19 pandemic highlighted an urgent need for PoC methods to detect SARS-CoV-2, including its variants. To address this, QuantuMDx initially developed a laboratory-based multiplex assay with simple-to-use lyophilised reagents that are stable when stored at room temperature, and quicker to run than many others^3^. However, these tests require automated equipment and batch testing, and cannot give rapid results for individual assessment.

QuantuMDx therefore repurposed cassettes, the portable analytical platform and software to provide an accurate, affordable and simple-to-use PoC test for SARS-CoV-2, as detailed in the Methods. Here it is demonstrated that, using a reference test Ct cut-off of 35, the Q-POC test for SARS-CoV-2 had a sensitivity of 96.88% (83.78–99.92% CI) and a specificity of 98.3% (82.2–99.9%). These performance characteristics are comparable to or better than other PoC NAAT devices^10–13^.

Centralised RT-PCR testing has the advantage of higher throughput capability with Q-POC being able to process only one cartridge at a time. This can be mitigated to an extent depending on the clinical setting and clinical testing algorithm by using multiple processing units with random access for each. An advantage of the Q-POC includes a rapid run-time (32 min) when compared with the longer running times of laboratory-based diagnostic platforms and some PoC NAAT devices also^10,11^. A possible disadvantage is the need to add the swab to a buffer and a following pipetting step to introduce the sample into the cassette, rather than placing the sample swab directly into the cassette. Given that MSwab does not inactivate viruses, this may require the use of a safety hood for sample flow in a laboratory context, although in non-laboratory contexts PoC devices including lateral flow tests have been used globally without requiring specialised handling facilities. The flexibility in sourcing samples into a buffer before processing retains the ability to re-test on the same instrument, the ability to retest on a different instrument/platform and to sequence retained samples. These are advantages of the described Q-POC/Q-CAS system that has been developed as a platform diagnostic.

Within this study, the Q-POC test failure rate was 5.2% (8/155). Of the 8 invalid results all were QC errors where 2 were invalid as the RNase P target was detected at > 40, 3 were invalid as the RNase P target was not detected at all, and 3 were invalid because both the internal process control (IPC; detected in the ROX channel) and the RNase P target were not detected. Consistent with improvements in manufacturing, following this preliminary evaluation the cartridge failure rate (‘invalid result’ category) is expected to be < 2% and within specifications and industry standards.

Ct values as an estimate of viral load and/or infectiousness can be influenced by many variables such as the site and methods for sample collection, the choice of diagnostic targets within the viral genome and sample quality^14^. In establishing cut-off values a key principle is that the clinical risk of transmission must be minimised, so that chains of transmission are effectively interrupted. Several reports have suggested a Ct cut-off for reference RT-PCR tests of < 35^15–17^. When Ct values are assessed in relation to the ability to culture virus, there are < 3% culture positive samples with Ct = 35^18^. This Ct cut-off value is the most clinically relevant, because samples with higher cut-off Ct values are non-infectious and therefore could be termed ‘biological false positives’ despite being frequently detected (with up to 50% of positive results in assays having higher Ct cut-off values)^19^. Interestingly, the reference test’s analytical sensitivity data demonstrate that a Ct of 35 equates to approximately 10 genome copies per reaction (Supplementary Date S4). If as the data suggest, the Q-POC test can detect similar viral loads but no lower, this would equate to a lower limit of detection (LLOD) of 200 copies/ml (with a 50 µl reaction volume in the cassette), which is in keeping with data in the IFU and a recently published study from Spain that evaluated the Q-POC test and estimated a LLOD of 100 copies/ml^20^. In this latter study, a cut-off Ct of 30 was suggested at which the sensitivity of the Q-POC test was 100% and in line with Spanish guidelines^20^.

At a reference test cut-off Ct of 35, the Q-POC test has excellent performance characteristics, that can be further examined in larger clinical trials. Testing strategies can be optimised for individual use cases, such as for travellers, health-care workers, prison services, triage of patients coming to emergency departments, wherever there is no laboratory and for contacts at risk of infection by timing testing strategies appropriately and by repeating testing at intervals when indicated.

Our study has several limitations. Although the sample size was small, it has been intended to assess and optimise performance of this novel technology not to develop individual use cases. Secondly, paired swab samples were not used for the reference and the Q-POC test rather both tests were run from a single swab sample. While this might remove the variable associated with multiple sampling from a participant subject at any given time point, the samples were placed into 3 ml of MSwab buffer (required for the Q-POC test) rather than the normal 1 ml of viral transport medium used in general for laboratory-based RT-PCR reference tests. This may have the effect of decreasing the reference test sensitivity by a small margin. Thirdly, the samples collected from the COVID-19 patients were not accordance with the IFU for the Q-POC test, which uses MN swabs. Further comparative studies would be needed to determine if NT swabs can be used without loss of sensitivity, although it is worth noting that a small study found no difference in diagnostic sensitivity when comparing NT and nasopharyngeal swabs^21^.

## Supplementary Information

Supplementary Information 1.

Supplementary Information 2.

Supplementary Information 3.

Supplementary Information 4.

## Data Availability

The datasets generated during and/or analysed during the current study are available in the supplementary information.
