# Supplementary material for: The QuantuMDx Q-POC SARS-CoV-2 RT-PCR assay for rapid detection of COVID-19 at point-of-care: preliminary evaluation of a novel technology
Source: Sci Rep. 2023 Jun 17;13:9827. doi: 10.1038/s41598-023-35479-9 (PMC10276817; doi:10.1038/s41598-023-35479-9)
Supplement: Supplementary file 3 — Supplementary Information 3. [file 41598_2023_35479_MOESM3_ESM.pdf]

S3 File. Q-POC™ performance characteristics (analytical sensitivity).

The Limit of Detection (LoD) or analytical sensitivity was determined as the lowest concentration of SARS-CoV-2 target, that could be detected by the Q-POC™ SARS-CoV-2 Assay with a ≥95% positivity rate.

SARS-CoV-2 Copies/mL	Replicates	Average Ct	Standard Dev.	RNase P Copies/mL	Average Ct	Standard Dev.	Sensitivity
1,000	5/5	41.89	0.80	500	34.11	0.32	100%
800	4/5	42.51	2.42	500	33.99	0.97	80%
600	4/5	43.00	0.76	500	33.94	0.47	80%
Negative	0/5	NA	NA	500	34.08	1.11	NA

Presumptive LoD determined by amplification of different concentrations of SARS-CoV-2 RNA in the presence of a fixed concentration of RNase P.

The LoD was verified by running 21 replicates below, at and above the LoD determined above. The data are presented in the table below and demonstrate a sensitivity of ≥95% at the LoD (1,000 Copies/mL SARS-CoV-2).

SARS-CoV-2 Copies/mL	Replicates	Average Ct	Standard Dev.	RNase P Copies/mL	Average Ct	Standard Dev.	Sensitivity
1,200	21/21	40.65	1.75	500	34.47	0.52	100%
1,000	20/21	41.89	1.64	500	35.57	1.25	≥95%
800	19/21	42.69	1.37	500	35.09	0.83	<95%
Negative	0/10	NA	NA	500	35.20	1.75	NA

Taken from the Q-POC™ SARS-CoV-2 Assay Instructions for Use (Version Issued July 2021).
