# Supplementary material for: The QuantuMDx Q-POC SARS-CoV-2 RT-PCR assay for rapid detection of COVID-19 at point-of-care: preliminary evaluation of a novel technology
Source: Sci Rep. 2023 Jun 17;13:9827. doi: 10.1038/s41598-023-35479-9 (PMC10276817; doi:10.1038/s41598-023-35479-9)
Supplement: Supplementary file 4 — Supplementary Information 4. [file 41598_2023_35479_MOESM4_ESM.pdf]

S4 File. QuantuMDx SARS-CoV-2 RT-PCR Detection Assay performance characteristics (analytical sensitivity).

The Limit of Detection (LoD) or analytical sensitivity was determined as the lowest concentration of SARS - CoV-2 target, that could be detected by the QuantuMDx SARS-CoV-2 RT-PCR Detection Assay with a ≥95% positivity rate. All samples underwent extraction as well as amplification and detection on the CFX96 Dx.

SARS-CoV-2 Copies	Replicates	Average Ct	Standard Dev.	Human RNA Concentration	Average Ct	Standard Dev.
1,000,000	3/3	19.54	0.24	25ng	25.45	0.79
100,000	3/3	22.96	0.12	25ng	26.45	0.23
10,000	3/3	26.18	0.39	25ng	28.03	0.47
1,000	3/3	29.44	0.12	25ng	28.24	0.53
100	3/3	32.44	0.32	25ng	28.62	0.88
10	3/3	36.92	0.27	25ng	28.91	0.12
1	1/3	43.81	NA	25ng	29.04	0.34
0.1	0/3	NA	NA	25ng	28.78	0.03
NTC	0/3	NA	NA	25ng	25.45	0.79

Presumptive LoD determined by triplicate amplification of SARS-CoV-2 RNA in multiplex with a fixed concentration of human RNA.

LoD was verified by running 25 replicates from extraction through to detection on Bio-Rad CFX96™ Dx. The data are presented in the table below and demonstrate detection of 25/25 samples.

Input	Copies/Reaction	Average Ct	Standard Dev.	Total Detected
SARS-CoV-2	10	35.20	0.43	25/25

Verification of presumptive LoD determined by 25 replicates of SARS-CoV-2 RNA at 10 copy input in multiplex with a fixed concentration of human RNA.

Taken from the QuantuMDx SARS-CoV-2 RT-PCR Detection Assay Instructions for Use (Version 11 Issued February 2022).
